# Supplementary material for: ALAN is a computational approach that interprets genomic findings in the context of tumor ecosystems
Source: Commun Biol. 2023 Apr 14;6:417. doi: 10.1038/s42003-023-04795-1 (PMC10104859; doi:10.1038/s42003-023-04795-1)
Supplement: Supplementary file 2 — Supplementary Information [file 42003_2023_4795_MOESM2_ESM.pdf]

Supplementary Figures and Legends.

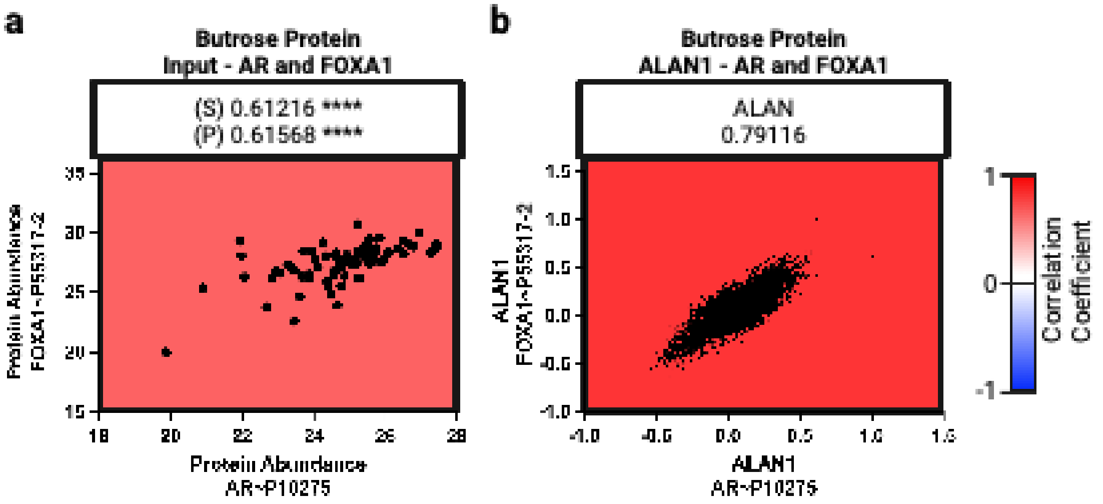

Supplementary Figure 1. The correlation between *AR* and *FOXA1* is depicted from blue (-1) to red (+1) in primary prostate cancer protein abundance data. **A.** Input matrix protein abundance data was used where each dot is a patient and its x- and y- coordinates represent the gene expression of *AR* or *FOXA1* respectfully and the Spearman's correlation was calculated. **B.** ALAN Output - Matrix 1 data was used where each dot represents an individual gene and its x- and y- coordinates represent the ALAN profile correlations with either *AR* or *FOXA1*.

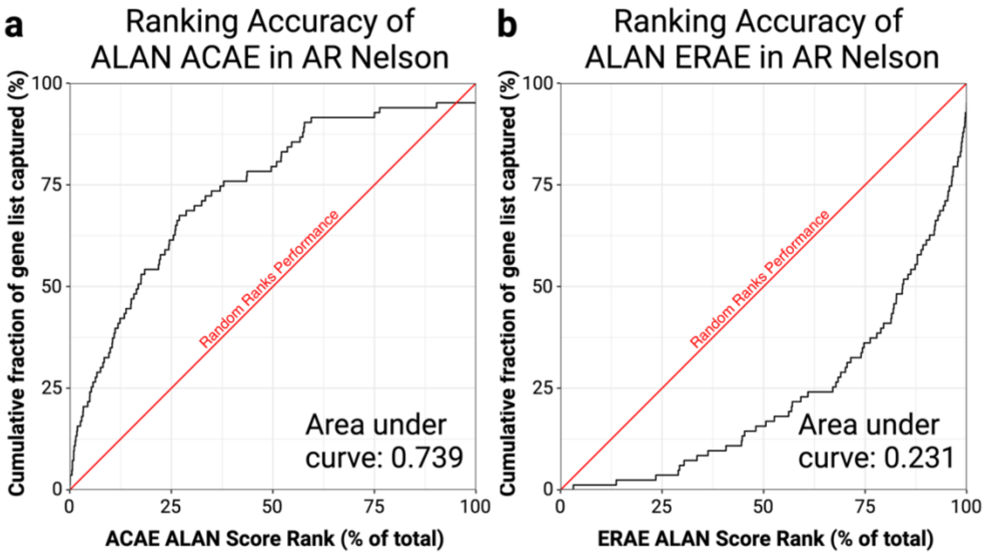

Supplementary Figure 2. Ranking Accuracy of ALAN Ecosystems against AR Nelson Gene Signature where performance against random ranks was measured by calculating the area under the curve (better > 0.5; worse < 0.5). **A.** The ACAE gene network represents the average gene networks of AR, FOXA1, and HOXB13 (ALAN Export Matrix 2). The resulting gene profile correlation coefficients were ranked and then the AR Nelson gene signature was scored. **B.** The ERAE gene network represents the average gene networks of CDK6, FGFR1, FGFR2, ETV5,

CREB5, and LEF1 (ALAN Export Matrix 2). The resulting gene profile correlation coefficients were ranked, and then the AR Nelson gene signature was scored.

Supplementary Tables.

Supplementary Table 1. Protein Interactions

**Gene1_Gene2**	**Spearman's Correlation**	**Pearson's Correlation**	**ALAN** Output Matrix 2
AR_FOXA1	0.49750 ****	0.64749 ****	0.91655
AR_HOXB13	0.43934 ****	0.64218 ****	0.89588

adj p-value > 0.05 (ns), adj p-value < 0.05 (*), adj p-value < 0.01 (**), adj p-value < 0.001 (***), adj p-value < 0.0001 (****)

Supplementary Table 2. Co-Regulators of the Same Pathway

**Gene1_Gene2**	**Cohort**	**Spearman's Correlation**	**Pearson's Correlation**	**ALAN** Output Matrix 2
AR_MYC	Normal Prostate	-0.18812 **	-0.24005 ***	-0.56905
AR_MYC	Primary Prostate	0.29229 ****	0.29106 ****	0.66999
AR_MYC	mCRPC	0.21240 ns	0.23552 *	0.72114

adj p-value > 0.05 (ns), adj p-value < 0.05 (*), adj p-value < 0.01 (**), adj p-value < 0.001 (***), adj p-value < 0.0001 (****)

Supplementary Table 3. Genes with Similar Function.

**Gene1_Gene2**	**Spearman's Correlation**	**Pearson's Correlation**	**ALAN** Output Matrix 2
FGFR1_CDK6	0.29616 ***	0.27019 **	0.82279
FGFR1_CREB5	0.27130 **	0.18176 ns	0.79487
CDK6_CREB5	0.32726 ****	0.21271 ns	0.82464
AR_CDK6	-0.18119 ns	-0.17542 ns	-0.75901
AR_CREB5	-0.28005 **	-0.24553 *	-0.76170
AR_FGFR1	-0.27272 **	-0.26861 **	-0.78961

adj p-value > 0.05 (ns), adj p-value < 0.05 (*), adj p-value < 0.01 (**), adj p-value < 0.001 (***), adj p-value < 0.0001 (****)
