# Supplementary material for: ALAN is a computational approach that interprets genomic findings in the context of tumor ecosystems
Source: Commun Biol. 2023 Apr 14;6:417. doi: 10.1038/s42003-023-04795-1 (PMC10104859; doi:10.1038/s42003-023-04795-1)
Supplement: Supplementary file 3 — Description of Additional Supplementary Files [file 42003_2023_4795_MOESM3_ESM.pdf]

Description of Additional Supplementary Files

File name: Supplementary Data 1
Description: The source data behind the graphs in the paper.
